# Concurrent multiple health risk behaviors among adolescents in Luangnamtha province, Lao PDR

**DOI:** 10.1186/1471-2458-11-36

**Published:** 2011-01-13

**Authors:** Vanphanom Sychareun, Sarah Thomsen, Elisabeth Faxelid

**Affiliations:** 1 Faculty of Postgraduate Studies, University of Health Sciences, Vientiane, Lao PDR; 2 Division of Global Health (IHCAR), Department of Public Health Sciences, Karolinska Institutet, Stockholm, Sweden; 3 InDevelop-IPM, Stockholm, Sweden

## Abstract

**Background:**

Multiple health risk behaviors (HRBs) among adolescents pose a threat to their health, including HIV/AIDS. Health risk behaviors such as alcohol use, smoking, substance use, and sexual risk behaviors among youth have been shown to co-occur with each others. The objectives of this study was to estimate the prevalence of single and concurrent health risk behaviors and to explore how health risk behavior is associated with socio-demographic factors and peers' behaviors.

**Methods:**

A cross sectional design was used to examine health risk behaviors of adolescents between the age 14 and 19 years living in the Luangnamtha province, Lao PDR. The study was conducted between June and August, 2008. An ordinal logistic regression model that simultaneously explored demographic factors and the influence of the behavior of peers on three categories of multiple HRBs (no risk, one risk, and two or more health risk behaviors) was performed.

**Results:**

A total of 1360 respondents, 669 (49.1%) boys with mean age 16.7 ± 1.6 and 699 (50.9%) girls aged 16.1 ± 1.5 were recruited into the study. The majority reported two or fewer risk behaviors. However, multiple risk behaviors increased with age for both sexes. About 46.8% (n = 637) reported no risk, 39.3 percent (n = 535) reported one risk, 8.1 percent (n = 110) reported two risks, and 5.8 percent reported more than two health risk behaviors.

The protective factors among boys were school attendance (OR = .53, CI = .33-.86), being Hmong and Yao ethnicity (OR = .48, CI-.26-.90), while being above the age of 15 (OR = 2.20, 95% CI = 1.33-3.60), Akha ethnicity (OR = 2.20, 95% CI = 1.04-4.61), peer's smoking (OR = 3.11, 95% CI = 2.1-4.6), and peer's drinking alcohol (OR = 1.88, 95% CI = 1.1-3.21) were significantly associated with the presence of multiple risk behaviors among boys.

Having some education (OR = 0.17, 95% CI = 0.06-0.45), and being of Hmong and Yao ethnicity (OR = 0.38, 95% CI = 0.18-0.80) were factors that protected girls from multiple risk behaviors; while peer's drinking alcohol (OR = 2.55, 95% CI = 1.59-4.09) and peer's being sexually active (OR = 2.82, 95% CI = 1.65-4.8) were significantly associated with the presence of multiple risk behaviors among girls.

**Conclusion:**

There are sex, age and ethnic differences in the concurrent health risk behaviors. The influencing factors are adolescent's education and peer influence. Interventions should focus to encourage adolescents to complete the compulsory primary education as well as help them to establish friendships and follow peers with good behavior. Risk reduction messages need to take account of diverse multiple HRBs within the specific socio-cultural and gender specific context and target vulnerable adolescents such as ethnic minorities and less educated adolescents.

## Background

Adolescents often engage in risky behaviors such as smoking, drinking alcohol, using drugs, and early unprotected sexual activity [1]. Risky behaviors might pose a threat to adolescents' future health. The adverse health consequences of these behaviors have been recognized as important public health issues [2,3]. When adolescents take one risk, they also tend to take other risks [4-9]. The interrelationship or cluster of health risk behaviors can be labeled as "risk behavior syndrome". This occurs in different combinations in different subpopulations [10-12].

The conceptual framework developed by Jessor [13] suggests that in the assessment of adolescent risk behavior, demographic-, socio-psychological- and environmental risk factors should also be considered. Concurrent predictors of health risk behaviors include the personality but also the biological factors, the family situation, and peer influence [14-17].

The population of the Lao PDR is 5.6 million and consists of 49 officially recognized ethnic groups, which have their own customs and languages. About 27.2 percent of the population lives in urban areas and 72.8 percent in rural areas [18]. Luangnamtha province is more rural (78%) and is populated by more ethnic groups than other parts of the country. These groups include Khamu, Akha, Hmong, and Yao (Mien). Besides this, the province also consists of low land Lao, Tai Lue, Tai Neua, and Tai Dam ethnic groups [19]. Different ethnic groups have different health risk behaviors. For example the Khamu has a higher prevalence of smoking compared to other ethnic groups in Laos [20]. Furthermore, members of the ethnic group Akha are teaching sexual practices to adolescents when they reach puberty and certain traditional sexual customs like *"Bong Hu"*, which means "open the vagina" are practiced. Another custom *"Hub Khet"*, which means to "Welcome Guest ", is also practiced in this ethnic group and means that the village head offers guests to sleep with girls from the village [21]. Thus, adolescents from different ethnic groups may be vulnerable to health risks such as substance use, but also the risk of being infected with sexual transmitted infections (STIs) and HIV because of unsafe sexual behavior.

Most previous studies have concentrated on risk behaviors of urban adolescents and not much is known about adolescents living in rural areas and especially not about those from ethnic minorities. For Lao adolescents, this gap in the understanding of their behavior is particularly important, since adolescents from rural areas and ethnic minorities are marginalized and more vulnerable than urban adolescents [22]. Previous research almost exclusively focused on single risk factors while adolescents are displaying a concurrent multiple health risk behavior, which is poorly understood especially in low-income countries. The aim of this study was to estimate the prevalence of single and concurrent health risk behaviors and to explore how health risk behavior is associated with socio-demographic factors and peers' behavior in order to get a better view of the constellation of multiple risk behavior factors among ethnic minorities in northern Lao PDR.

## Methods

### Setting

Luangnamtha province is located in the northern part of Lao PDR. The province has a population of approximately 145,000 people with a sex ratio male to female of 1:1.04 and 78.2% of population is living in rural areas. The economic bases are agricultural and tourism. The adult literacy rate (among those age 15 and above) is the lowest in the country, 32.5 percent, versus 60.2 percent for the whole country. Literacy of boys in comparison to girls is 50.6 percent vs 43.1 percent. Three percent of girls and five percent of boys have completed upper secondary school, which is lower than the national figures (10% for girls and 12% for boys) [18].

Until recently, the Luangnamtha province ranked third for prevalence of HIV in the country. The Yunnan Province of the People Republic of China is next to the Luangnamtha province and the province is an entry point for international drug trafficking. Tourism has increased mobility of the population and has aggravated the HIV/AIDS pandemic. In the highlands of the Sing and Long districts, opium has been grown extensively to be used both as an effective medicine for a range of illnesses and a crucial source of income, especially in exchange for rice in times of food shortage [21].

#### Study design and Participants

A cross sectional study design was applied to examine the selected health risk behaviors of adolescents between the age 14 and 19 years in the Luangnamtha province. The study was conducted between June and August, 2008.

Four out of the five districts in the province were randomly selected. A list of villages for each selected district was obtained from the district governance. A two stage sampling procedure was followed. Firstly, from the list of villages of the four districts, 64 out of 294 villages were randomly selected with proportional to size; the bigger the target population size in the district, the more villages within the districts were selected. Secondly, from each selected village, unmarried male and female adolescents aged 14-19 years were listed based on the household census booklet, which was verified and updated by the head of villagers. These adolescents were systematically randomly selected from the constructed sampling frame with probability proportional to the size of the villages. If there was more than one boy and girl in the selected household, one of each sex was randomly selected.

A face-to-face interview with the selected adolescents was conducted to obtain the required information. If the identified respondent was not available at the time of the visit, appointments were made to return for the interview at least twice. About 97.2 percent of all eligible boys and girls participated.

#### Measures of socio-demographic factors

Data on the socio-demographic characteristics of adolescents and their families included age, ethnicity, education, currently attending school, living arrangements, source of income and whether income was enough for expenses, parent's marital status, parent's education, parental occupation, and source of sexuality education. The current school enrollment was defined as either "in school" (attending school or on vacation from the school at the time of interview) or "out-of-school" (not attending school at the time of interview). Parental information was based on asking adolescents about their father's and mother's highest completed level of education. Parent's education level was classified as illiterate, primary, secondary, vocational and higher educational level. The categories of secondary, vocational and higher educational level were combined to one such as secondary and higher due to small denominators.

#### Measures of the health risk behaviors

The selected health risk behaviors (HRBs) were assessed by asking the adolescents whether they had smoked cigarettes and had drank alcohol at least one day during the previous month, took amphetamine tablet in the past 30 days prior to the survey, had multiple sex partners during the past six months, first sexual intercourse before the age of 15, and not using condoms during last sexual intercourse.

These measures were used to determine the prevalence of the single health risks among the participants. For the prevalence of concurrent health risks, only current alcohol use, current smoking, currently being sexually active, first sexual intercourse before age 15 years and lack of condom use during last sexual intercourse were included. Subsequently, subjects were classified as having none, one, and two or more HRBs. Amphetamine use and having more than one sexual partner during the last six months were not included in the multiple concurrent HRBs due to low prevalence, in particular among girls.

The participants' perceptions of "peer involvement in risk behaviors" were assessed for three items related to peer substance use behavior: drinking alcohol, smoking, and using drugs. Peer sexual behaviors such as whether friends had became pregnant or caused someone to become pregnant, and whether she or he was sexually active were also assessed. The responses related to peer's behavior were dichotomized as yes or no.

#### Data collection

The interviewers, who were from the Faculty of Postgraduate Studies (young graduated medical doctors) and from the district health office (medical assistants), were trained in the purpose of the study, the content of the questionnaire and how to conduct the interview. The interviewers were matched with the sex of interviewees. For respondents who spoke the local language a translator was used. Interviews were carried out in privacy and the respondents were assured anonymity and confidentiality. Verbal consent of the parents was obtained for adolescents less than 18 years, whereby parents were notified that their child was invited to participate in an anonymous survey. The purpose of the survey was explained to the participants, and they were given the option to participate or withdraw or skip questions they were not comfortable to answer. The study was approved by the National Ethics Committee Board for Health Research, Ministry of Health, Lao PDR with Ref: 175 NECHR, dated 25^th ^June, 2008.

#### Data analysis

Chi-square analyses were performed to determine the association between the socio-demographic background and peer's health risk behaviors and the three categories of multiple HRBs (no risk, one risk, and two or more health risk behaviors). The independent variables were entered into a comprehensive logistic regression model that simultaneously considered the demographic and peer health risk behaviors. SPSS statistical software was used to perform ordinal logistic regression analysis for co-occurrence of multiple health risk behaviors. This technique is similar to logistic regression, although, it allows for more than two ordinal outcome levels because the HRB variable had three outcome levels such as no risk, one risk, two or more risks. All analyses were performed separately for both sexes. All tests were two sides and statistically significant at the p-value <.05.

## Results

### Socio-demographic background

A total of 1,360 respondents, of which 669 were boys (49.1%) were recruited into the study. The mean age of the boys was 16.7 (SD = 1.6) and that of the girls was 16.2 years (SD = 1.5). About 68.5 percent of respondents were attending school during the time of the survey although the boys were more likely to attend school than the girls (72% vs. 65%, p <.01). The main source of income was from parents (85.6%), followed by earning money while working (41.7%) (Table 1).

**Table 1:** Socio-demographic characteristic of adolescents aged 14-19 years old and their parents in Luangnamtha province, Lao PDR

Variables	Total		Boys		Girls		p-value
	N = 1360	%	N = 669	%	N = 691	%	
**Age**							<.001
=<15	447	32.9	184	27.5	263	38.1	
16-19	913	67.1	485	72.5	428	61.9	
**Ethnicity**							.784
Lao	504	37.1	241	36.0	263	38.1	
Akha	249	18.3	123	18.4	126	18.2	
Khamu	435	32.0	215	32.1	220	31.8	
Hmong & Yao	172	12.6	90	13.5	82	11.9	
**Education**							<.001
Never	107	7.9	28	4.2	79	11.4	
Primary	402	29.5	196	29.3	206	29.8	
Secondary & higher	851	62.6	445	66.5	406	58.8	
**Attending school**							.005
Out-of school	429	31.5	187	28.0	242	35.0	
In-school	931	68.5	482	72.0	449	65.0	
Source of income*							
Working	567	41.7	244	36.5	323	46.7	<.001
Parents	1164	85.6	559	83.6	605	87.6	.037
Scholarship	18	1.3	12	1.8	6	0.9	.158
Relative	166	12.2	95	14.2	71	10.3	.031
Others	92	6.8	10	1.5	82	11.9	<.001
**Living arrangement**							.013
Other	244	17.9	137	20.5	107	15.3	
Family	1116	82.1	531	79.5	585	84.7	
**Father's education**							.107
Illiterate	334	31.0	163	29.5	171	32.8	
Primary	480	44.7	242	43.7	238	45.7	
Secondary & higher	261	24.3	149	26.9	112	21.5	
**Mother's education**							.442
Illiterate	674	57.2	335	56.8	339	57.6	
Primary	388	32.9	190	32.2	198	33.6	
Secondary & higher	117	9.9	65	11.0	52	8.8	

Note:

N for mother's education = 1179 due to separated/divorced and death

N for father's education = 1075 due to separated/divorced and death

Chi-square was used to test the difference between boys and girls and Fisher's exact

test was used, when the expected value is less than 5 more than 20%.

* This is the multiple responses and the percentages were calculated based on the

number of respondents, the sum of responses therefore may be ≥100%

More girls than boys lived with their parents (84.7% vs. 79.5%, p = 0.013). The educational level of the parents in general was low but the fathers were better educated than the mothers. Slightly less than half (44.7%) of respondent's fathers had completed primary school, while 24.3 percent of the fathers had finished education above primary. About 32.9 percent of the mothers had a primary school education and 9.9 percent a secondary or higher education (Table 1).

### Health risk behaviors

Health risk behaviors are shown in Table 2. Percentages for risky behaviors are higher in the older group for both boys and girls than the younger group, with the exception of those sexually active during the last six months, having first sex before 15 years and not using of condoms. For the younger age group, 14-15 years old, the most common single risk behavior was currently being sexually active, having the first sexual experience before the age of 15 years and not using condoms during the last sexual intercourse. Other health risks were less common. For the age group 16-19 years old, 49.8 percent drank alcohol, 12.8 percent smoked cigarettes, 55.7 percent had sex during the last six months, 44.1 percent had multiple sex partners during the last six months, and 51.7 percent did not use a condom during the last sexual intercourse.

**Table 2:** Prevalence of health risk behaviors among adolescents by age and sex in Luangnamtha province, Lao PDR

	14-15 years		P- value	16-19 years		P- value
	N = 447	%		N = 913	%	
**Current alcohol use**	123	27.5		455	49.8	
Boys	39	21.2	.013	266	54.8	.001
Girls	84	31.9		189	44.2	
**Current cigarette use**	14	3.1		117	12.8	
Boys	14	7.6	<.001	114	23.5	<.001
Girls	0	0		3	0.7	
**Current amphetamine use**	1	0.2		12	1.3	
Boys	1	0.5	.412	12	2.5	.001
Girls	0	0		0	0	
**Having sex during the last 6 months+**	39	70.9		118	55.7	
Boys	23	71.9	1.000	92	54.1	.391
Girls	16	69.6		26	61.9	
**Age at first sex before 15 years+**	39	70.9		47	22.2	
Boys	25	78.1	.231	35	20.6	.300
Girls	14	60.9		12	28.6	
**Two or more partners during the last six months**	11	28.2		52	44.1	
Boys	9	39.1	.086	48	52.2	.001
Girls	2	12.5		4	15.4	
**Not using condoms for the last sexual intercourse++**	27	69.2		61	51.7	
Boys	14	60.9	.40	47	51.1	.072
Girls	13	81.3		14	53.8	

Note:

+ among those who ever reported having had sex

++ among those who were sexually active during the last 6 months prior the

survey

Chi-square was used to test the difference between boys and girls for the

younger and older adolescents and

Fisher's exact test was used when each cell has an expected value less

than 5 more than 20%.

More boys than girls reported having two or more sexual partners during the last six months. However, there was an age difference as more of the older respondents reported two or more sexual partners. At 16-19 years, a lower proportion of both sexes reported not using condoms, but the difference between the sexes was not significant (51.1% vs. 53.8%).

### Co-occurrence of multiple health risk behaviors

Of the 1,360 subjects, 46.8 percent reported no risks, 39.3 percent reported one risk, 8.1 percent reported two risks, and 4.2 percent (n = 57) reported three, 1.2 percent (n = 16) reported four, and 0.4 percent (n = 5) reported five risk behaviors (Table 3). The risk behaviors of the adolescents tend to fall into specific patterns. For the boys who had two risk behaviors, the most common two risk behaviors were alcohol use and smoking, followed by being sexually active and not using condoms. For girls who had two risk behaviors, the most common two risk behaviors were being sexually active and not using condoms. Boys were more likely to report two health risk behaviors than girls (11.1% vs. 5.1%, p <.001). A small percentage of adolescents (more boys than girls) involved in more than two health risk behaviors (Table 3).

**Table 3:** Patterns of health risk behaviors among adolescents 14-19 years, in Luangnamtha province, Lao PDR

	Within category		
	**Male** **(n = 669)**	**Female** **(n = 691)**	**P- value**

**No risk**	-	-	<.001
**One single risk**	-	-	
Alcohol use	45.6	39.5	.025
Current smoking	19.1	0.4	<.001
Currently sexual active	56.9	64.6	.312
Having first sexual intercourse before 15 years	29.7	40	.129
Not using condoms during last intercourse	63.2	72.3	.231
**Two risks**			
Alcohol drinking & smoking	14.9	0.3	<.001
Alcohol drinking & sexual active	9.4	2.0	<.001
Alcohol drinking & first sex before 15 years	2.8	0.9	<.001
Alcohol drinking & not using condoms	9.4	2.5	<.001
Smoking & sexual active	5.7	0.1	<.001
Smoking & first sex before 15 years	2.2	0	<.001
Smoking & not using condoms	7.2	0.1	<.001
Sexually active & first sex before 15 years	5.4	2.5	<.001
Sexually active & not using condom	9.6	4.2	<.001
**Three risks**			
Alcohol drinking, smoking, & sexually active	5.5	0.1	<.001
Alcohol drinking, smoking, & first sex before 15 years	1.5	0	<.001
Alcohol drinking, smoking, & not using condoms	5.5	0.1	<.001
Sexual active, first sex before 15 years & not using condoms	3.6	1.6	<.001
**Four risks**			
Alcohol drinking, smoking, sexual active & not using condoms	3.0	0.1	<.001
Alcohol drinking, smoking, sexual active & first sex before 15 years	1.2	0	<.001
**Five risks**			
Alcohol drinking, smoking, sexual active, first sex before 15 years & not using condoms	0.9	0	<.001

Chi-square was used to test the difference between boys and girls for the

younger and older adolescents and

Fisher's exact test was used when each cell has an expected value less

than 5 more than 20%.

Table 4 and 5 give the results of bivariate and multivariate analyses of multiple health risk behaviors by socio-demographic conditions and peer influence. The majority of adolescents had none or only one health risk. However, the prevalence of multiple risks increased with age for boys, but not for girls. Out-of-school adolescents of both sexes were more likely to have concurrent health risk behavior than in-school adolescents (p<.001). For both age groups, boys outnumbered girls in concurrent health risk behaviors.

**Table 4:** Bivariate analyses of concurrent multiple health risk behaviors among boys and adolescents by socio-demographic backgrounds and peer influence

Variable	Boys					Girls				
	N	No risk	One risk	Two or more	P-value	N	No risk	One risk	Two or more	P-value
		%	%	%			%	%	%	
**Age**					<.001					.002
14-15	184	62.5	24.5	13		263	62.4	30.4	7.2	
16-19	485	29.7	46.0	24.3		428	49.8	43.7	6.5	
**Attending school**					<.001					<.001
Out-of school	187	24.1	43.3	32.6		242	44.2	40.9	14.9	
In school	482	44.4	38.8	16.8		449	60.1	37.4	2.4	
**Adolescent's education**					<.005					<.001
Illiterate	28	28.6	42.9	28.6		79	43.0	29.1	27.8	
Primary	196	43.9	29.6	26.5		206	54.9	34.0	11.2	
Secondary & others	445	37.1	44.5	18.4		406	56.7	42.9	0.5	
**Ethnicity**					<.001					<.001
Lao	241	36.5	43.6	19.9		263	51.3	48.3	0.4	
Akha	123	43.1	26.8	30.1		126	52.4	23.8	23.8	
Hmong & Yao	90	63.3	28.9	7.8		82	76.8	19.5	3.7	
Khamu	215	28.4	48.4	23.3		220	51.4	42.7	5.9	
**Living arrangement**					.338					.582
Other	138	33.3	44.2	22.5		106	53.8	41.5	4.7	
Family	531	40.1	39.0	20.9		585	54.7	38.1	7.2	
**Father's education**					.014					<.001
Illiterate	163	46.0	28.8	25.2		171	50.9	32.2	17.0	
Primary	242	36.4	43.8	19.8		238	57.1	39.5	3.4	
Secondary & others	149	38.9	45.0	16.1		112	59.8	40.2	0	
**Mother's education**					.029					<.001
Illiterate	335	43.3	34.6	22.1		339	54.9	33.9	11.2	
Primary	190	34.2	48.9	16.8		198	53.5	45.5	1.0	
Secondary & others	65	41.5	41.5	16.9		52	53.8	46.2	0	
**Peers using alcohol**					<.001					<.001
No	179	63.1	20.1	16.8		226	73.5	18.1	8.4	
Yes	490	29.8	47.3	22.9		465	45.4	48.6	6.0	
**Peers smoking**					<.001					.009
No	375	52.3	33.9	13.9		636	56.0	37.9	6.1	
Yes	294	21.4	48.0	30.6		55	38.2	47.3	14.5	
**Peer using drug**					.008					.271
No	643	39.5	40.2	20.2		669	55.0	38.4	6.6	
Yes	26	15.4	42.3	42.3		22	40.9	45.5	13.6	
**Peers being sexually active**					<.001					<.001
No	279	49.5	40.1	10.4		586	58.4	36.9	4.8	
Yes	390	31.0	40.0	29.0		105	33.3	48.6	18.1	
**Peers have been pregnant**					.003					.132
No	625	40.2	39.8	20.0		647	55.5	38.0	6.5	
Yes	44	18.2	43.2	38.6		44	40.9	47.7	11.4	
Total	669	38.7	40.1	21.2		691	54.6	38.6	6.8	

**Table 5:** Multivariate analyses of concurrent multiple health risk behaviors among boys and girls by socio-demographic backgrounds and peer influence

Variable	Boys		Girls	
	
	Adjusted OR	95%CI	Adjusted OR	95%CI
**Age**				
14-15	1		1	
16-19	2.2	1.33 - 3.60	1.31	.84 - 2.03
**Attending school**				
Out-of school	1		1	
In school	0.53	.33 - .86	0.65	.40 - 1.06
**Adolescent's education**				
Illiterate	1		1	
Primary	1.44	.48 - 4.28	0.19	.08 - .46
Secondary & others	1.42	.47 - 4.32	0.17	.06 - .45
**Ethnicity**				
Lao	1		1	
Akha	2.2	1.04 - 4.61	0.88	.40 - 1.93
Hmong & Yao	0.48	.26 - .90	0.38	.18 - .80
Khamu	1.56	1.02 - 2.38	0.97	.61 - 1.54
**Living arrangement**				
Other	1		1	
Family	0.94	.49 - 1.82	1.63	.69 - 3.86
**Father's education**				
Illiterate	1		1	
Primary	1.27	.77 - 2.10	0.77	.45 - 1.29
Secondary & others	1.24	.70 - 2.20	0.61	.32 - 1.16
**Mother's education**				
Illiterate	1		1	
Primary	0.92	.59 - 1.41	0.92	.56 - 1.48
Secondary & others	0.72	.37 - 1.39	0.97	.46 - 2.03
**Peers using alcohol**				
No	1		1	
Yes	1.88	1.10 - 3.21	2.55	1.59 - 4.09
**Peers smoking**				
No	1		1	
Yes	3.11	2.10 - 4.60	1.83	.89 - 3.73
**Peer using drug**				
No	1		1	
Yes	1.9	.77 - 4.67	1.72	.59 - 5.02
**Peers being sexually active**				
No	1		1	
Yes	1.31	.90 - 1.92	2.82	1.65 - 4.8
**Peers have been pregnant**				
No	1		1	
Yes	1.87	.93 - 3.77	0.89	.341 - 1.96

Note: Odds ratios (OR) were calculated using an ordinal logistic regression analysis. Categories of health risk behaviors (HRB: none, one, two or more) were mutually exclusive. Odds ratios reflect one-unit change in the independent variables.

In the bivariate analysis, for boys, age above 15 years and the ethnicity Akha were significantly associated with multiple health risk behaviors. Boys who had peers with health risk behaviors were more likely to have multiple risk behaviors than boys whose peers had no health risk behavior. Boys with a higher level of education, currently attending school, and having parents with some level of education were negatively associated with multiple health risk behaviors. For girls, older age, attending school, higher level of education, Khamu, Hmong and Yao, and Lao ethnicities, and parents having some level of education were associated with fewer risk behavior, while peer's involvement in health risk behaviors such as smoking, drinking alcohol and having sex were associated with multiple risk behaviors.

In the multivariate analysis, attending school (OR = .53, 95% CI = .33-.86), Hmong and Yao ethnicity (OR = .48, 95% CI = .26-.90) were the protective factor for multiple risk behaviors, while older age (OR = 2.20, 95% CI = 1.33-3.60), Akha ethnicity (OR = 2.20, 95% CI = 1.04-4.61), peers smoking (OR = 3.11, 95% CI = 2.1-4.6), and peers drinking alcohol (OR = 1.88, 95% CI = 1.1-3.21) were significantly associated with multiple risk behaviors among boys. For girls, having some education (OR = 0.17, 95% CI = 0.06-0.45) and being of Hmong and Yao ethnicity (OR = 0.38, 95% CI = 0.18-0.80) were significantly associated with fewer risk behaviors; while peers perceived drinking alcohol (OR = 2.55, 95% CI = 1.59-4.09) and peers being sexually active (OR = 2.82, 95% CI = 1.65-4.8) were associated with multiple risk behaviors.

## Discussion

To our knowledge this is the first study investigating the prevalence of concurrent HRBs among adolescents and examining the associations between the risk factors for multiple HRBs in Lao PDR. Although it seems that few adolescents engaged in two or more concurrent HRBs, it is important to notice that overall risk-taking among adolescents is rather common, in particular among adolescents from minority ethnic groups.

The study revealed that more boys than girls drank alcohol and smoked tobacco in the older age group, which is consistent with findings from other studies conducted in South East Asia [23-25]. Our study, however, detected higher rates of alcohol drinking among girls in younger age groups compared with boys of the same age group. The reason might be that girls are more likely to socialize with and being persuaded by friends to drink alcohol compared to boys, which was also reflected in the association between own risk behavior and peers drinking alcohol. It has also been shown elsewhere that adolescents exposed to high levels of alcohol availability are more likely to drink alcohol than adolescents not so exposed [26]. An alternative explanation is that younger boys may drink alcohol equally or more than younger girls, but they are less likely to report. Girls in the younger age group might be more vulnerable to the aftereffects of alcohol drinking than their male counterparts because of girls' lower body-weight [27]. The higher rates of drinking alcohol, smoking, and using amphetamine among males in the older age group are dangerous since the habit often continues into adulthood [2,28].

Early age at first sexual intercourse (before 15 years) was higher among younger boys than girls as have been found in previous studies [29,30]. In Lao PDR, most adolescents in rural areas start to have sexual intercourse at early age, which is consistent with previous research in Lao PDR [31]. The alarming rate of sexual risk behaviors among the younger adolescents may be explained by their curiosity, experimentation with new things and hormonal change but also related to some sexual traditional customs among some ethnic groups in Luangnamtha province such as 'welcome guest', and 'open vagina'. This is consistent with previous research by Lyttleton et al [21], who also described these traditional sexual customs that are practiced in Lunagnamtha province. Research suggests that early sexual intercourse is associated with sexually transmitted infections [32] and early pregnancy, which might result in abortion and immature childbirth [33,34].

Sexual risk behaviors were more prevalent among boys than girls. Slightly more than half of older boys in this study had two or more sexual partners, which is consistent with previous research showing that about 70 percent of male students in the Republic of Korea and about 30 percent of young men in Thailand had two or more sexual partners [35]. The higher prevalence of two or more sexual partners among older adolescents could be explained by the fact that the older age group was more sexually experienced and they might also socialize with sexually experienced friends to a higher extent than the younger counterparts.

Most adolescents in our study did not use a condom during the last sexual intercourse. Low condom use among adolescents seems to be a trend in the region. A previous study in the Lao PDR indicated that 75 percent of sexually experienced adolescents aged 15 to 25 years did not use any contraceptive method at first sexual intercourse [36]. Similarly, a study carried out in Thailand also found that condom use during last sexual activity was 16 percent among sexually active males and 11 percent among sexually active female high school and vocational students [37].

Another important finding is the sex difference for risk factors for multiple HRBs. Boys seemed to be more likely than girls to engage in multiple risk behaviors. This might be due to cultural acceptance for young boys to engage in alcohol use, smoking, and sexual activity. The alternative reason is social desirability reporting bias in which boys may exaggerate and girls underreport their multiple health risk behaviors. Previous studies in China [38] show similar results with male students being more likely to report engaging in multiple risk behaviors than female students.

Our findings suggest that having some education and attending school are protective factors for both sexes considering multiple health risk behaviors. Out-of school adolescents belong to a vulnerable group for risk-taking and they are at higher risk of engaging in multiple health risks. The reason might be that they are more relaxed to socialize with many people compared to in-school youth who have more strict rules to observe. In Lao PDR, school regulations are strict and the behaviors of adolescents are controlled, which might limit health risk behaviors. Similar results have been found in the United States, Ethiopia, and China [11,39,40].

Multiple risk health behaviors differed significantly between ethnicities, being highest among Akha adolescents of both sexes and lowest among Hmong and Yao adolescents. However, not much is known about Asian ethnic groups as most studies focus on African, American or Causacian adolescents. The ability to differentiate between ethnic adolescents' is limited by the lack of anthropological data about health risk behavior of ethnic groups in Lao PDR as well as in other South East Asian countries. This study has shown that adolescents from certain ethnic groups are at risk of multiple health risk behaviors, which might be due to their cultural beliefs and practices related to smoking, drinking alcohol, and sexuality. Some of the adolescents in our study seemed to have sexual freedom and started having sex at an early age, which corresponded with prior research in Luangnamtha province [21].

Peer involvement in health risk behaviors seemed associated to adolescents' multiple health risk behaviors. Jessor and Jessor (1977) suggested that adolescents who are more connected with their peers than with their parents, especially peers with negative behaviors, were more likely to practice risky health-related behaviors [41]. In our study, peer behavior was associated with health risk behaviors among the participants such as smoking, alcohol use and sexual activity, which corresponds with previous studies in other parts of the world [17,42-46]. Blanton (2001) also suggests that peer influences were more likely to be manifested when the target and the peer shared similar behavioral histories, which is consistent with social comparison theories [47].

### Limitations

This study has some limitations that are worth mentioning. This investigation is a cross-sectional study and thus it is not possible to determine either causality or directionality of any health risk behaviors. Co-occurrence is not necessarily a proof that one behavior causes the other. Reported use of specific drugs were not provided because there were too few who used drugs, then the estimates would have been too imprecise. Since we rely on self-reporting a potential bias caused by the questioning format and the sensitive nature of some health risk behaviors is possible. Some behaviors might thus be over reported by boys and under-reported by girls. However, there are no sexual taboos in some ethnic groups as they have some sexual ritual practices during the puberty of adolescents. We try to reduce the bias by matching the sex between interviewers and respondents.

## Conclusion

This study highlighted that there are sex, age and ethnic differences in the concurrent risk behaviors. The most common concurrent risk behaviors among boys was alcohol use and smoking, followed by being sexually active and not using condoms, while alcohol use, sexually active, and not using condoms were the most common concurrent risk factor for the girls. The influencing factors on multiple HRBs are present in one's demographic background namely adolescent's education and peers influence.

### Implication

The findings have important policy implications. Addressing the co-occurring risk factors in public health settings might prevent future behavioural health risks. Strategies might be to encourage young people to finish school, be selective in choosing their friends, and aimed for health attitudes and behavior. Focus on the reduction of risk should be multifactorial within the specific socio-cultural and gender specific context. Tailoring behavior change interventions to individual needs and circumstances is also essential. Interventions should target vulnerable groups such as younger adolescents, ethnic minority groups, and out of school adolescents.

Further research is required to examine to what extent these findings can be replicated in other areas of Lao PDR, especially in respect of sexual risk behaviors among ethnic minority groups. In addition, there is a need to explore in depth factors influencing adolescent's sexualities such as their safe and unsafe sexual practices and what programs are most effective and efficient in addressing multiple risk behaviors among adolescents.

## Competing interests

The authors declare that they have no competing interests.

## Authors' contributions

SV developed the research proposal, designed the instrument, collected data in the field sites, ran data analysis, and drafted the manuscript. EF supervised the research project and assisted via expertise in the survey instrument development, data collection and data analysis. ST contributed to the study design and helped in improving the manuscript. All authors read and approved the final manuscript.

## Pre-publication history

The pre-publication history for this paper can be accessed here:

http://www.biomedcentral.com/1471-2458/11/36/prepub
